# Renal Disease—Probable Tape-Worm

**Published:** 1874-02-01

**Authors:** D. B. Trimble

**Affiliations:** Chicago, Ill.


### RENAL DISEASE—PROBABLE TAPE-WORM.

Two Cases From Practice by D. B. Trimble, M.D., Chicago, Ill.

THE following case may be of some interest to your readers, as showing the advantage of a careful diagnosis in a serious disease, before resorting to treatment.

Mr. S., a gentleman whose family had recently moved to the city, applied to me, late in November, to prescribe for his son, a child about two and a half years old, who had been in poor health for three or four months, and attended by two physicians, at different times.

They had pronounced his case indigestion or dyspepsia, and had treated him for this disease, but with little benefit. His father informed me that he had but little appetite; was considerably emaciated; very restless, especially at night, when he would have to rise to micturate very frequently, passing but little urine at a time, and that high-colored. He said that he had no pain in the bladder, apparently, but much irritability, making the inclination to urinate very urgent. From the symptoms, I suspected some renal difficulty, and before prescribing, requested him to bring some of the urine to analyze.

On the 29th, he brought me a four-ounce bottle full of very turbid urine, which, on standing about an hour, precipitated more than a quarter of an inch in depth of a pale red, or pink, deposit. The analysis showed the following condition:

Specific gravity, 1.030. Acid reaction, by litmus paper, very strong. The microscope developed the existence of amorphous urates and pus, very largely. Nitric acid showed a trace of albumen, but cleared the urine by dissolving the urates. On the application of heat, and the liquor potassæ, pus was precipitated. I prescribed for him, to meet the acid indication, aqua calcis; for the irritation, or inflammation of the mucous coat of the bladder, the following:

℞.—Ext. pareira brava, fld.
Ext. uva ursi, fld., aa gtt. xv.
Plumb. acet., gr. ½.

Three times a day.

My theory was, that the urates had induced the inflammatory condition of the bladder, and the formation of pus; and the indications were to neutralize the former, and subdue the latter.

On the 5th or 6th of December, a second portion of urine was brought me, with the information that there was some improvement in the symptoms. The urine presented the following conditions:

Deposit, fifty per cent. less. Specific gravity, 1.023. Acid reaction, strong, though somewhat diminished. Amorphous urates and pus still present, but in smaller quantities, as indicated by all the preceding tests. The only additional recipe that I now gave, was bicarbonate of soda, in five grain doses, three times a day.

On December 10th, I was requested to see him, and found him somewhat emaciated, pale, constipated, and with considerable fever. I prescribed a dose of ol. ricini, and spirits nitre, to be added to his alkaline solution.

Dec. 12.—Received the third specimen of urine. I was informed that he was much better; rested comparatively well; sometimes not being disturbed all night to micturate. Urine more plentiful, and less irritating; fever abating. The urine presented the following characteristics:

Urine, after standing for two hours, had no deposit. Specific gravity, 1,018. Slight acid reaction. Amorphous urates had nearly disappeared; perceived a few by microscope. No albumen. No deposit of pus from heat and liquor potassæ. Urine nearly normal.

I now discontinued the fluid extracts and acetate lead, except one dose at night, and discontinued the soda and nitre. Continued the aq. calcis.

On the 17th, examined the fourth and last specimen of urine. There was no deposit after two hours. Specific gravity, 1.015. Acid reaction, very moderate. Amorphous urates and pus disappeared. Urine normal. Omitted all the former course, and, as the little patient had a poor appetite, and was debilitated, gave him Sargent's elixir of the pyrophosphate of iron, and calisaya, with the $\frac{1}{120}$ grain strychnia, twice a day, under which treatment he is rapidly improving.

From reading your cases of tænia in the December 15th number of THE EXAMINER, I am induced to give you, very briefly, the following case, which I believe to have been tapeworm.

On October 5th, visited Mrs. D., a stout and fleshy young woman, who had grown fat rapidly. She was suffering with intense paroxysmal pains in her stomach, as she informed me, recurring every fifteen minutes. She had a high fever, a densely furred tongue, and constipation. Prescribed an active cathartic, to be followed by neutral mixture, and a combination of subnit. bismuth, and sulph. morph., alternating them.

On the 6th, found that the cathartic operated freely; the pain was mitigated for a few hours, but returned again in full force.

On the 7th, the fever had abated; the tongue commenced to clean; but the pain continued with the same intermitting symptoms.

I now began to suspect that it was neuralgia of the bowels (having before feared gastro-enteritis), and treated it for that disease, but with very slight alleviation. I did not see her again until the 9th, when I found her entirely free of fever, and the tongue nearly clean, but with a total loss of appetite. The pain, however, continued without any abatement, which she now referred to the umbilical region, and stated, that for two nights, she had "nearly choked to death," from "something rising in her throat." As she informed me, in reply to my questions, that she had, about a year before, passed "a white worm, about three feet long, and flat, and broken pieces about an inch long," I suspected another tape-worm. I therefore gave her the following: Pulv. pepo, ten drachms; ether. ext. filix mass, one drachm, made into an emulsion, of which she was to take one-fourth part every half hour, and to follow it by ol. ricini, one ounce; ol. terebinth., one drachm. It vomited her after the third drachm dose, and purged actively; but she saw no evidence of the worm. She was, however, entirely relieved of her pain, and her health has since been good, though she has lost some flesh.

Was it a tape-worm?

